# Relationship of Spiritual Intelligence With Resilience and Perceived Stress

**Published:** 2014

**Authors:** Masoumeh Khosravi, Zahra Nikmanesh

**Affiliations:** 1 Department of Psychology, School of Education and Psychology, University of Sistan and Baluchestan, Zahedan, Iran.; 2 Associate Professor, Department of Psychology, School of Education and Psychology, University of Sistan and Baluchestan, Zahedan, Iran

**Keywords:** Perceived Stress, Resilience, Spiritual Intelligence

## Abstract

**Objective:**

The purpose of this study was to investigate on relationship between spiritual intelligence, resilience, and perceived stress.

**Methods:**

The study sample consisted of 307 students of Sistan and Baluchistan University. The Connor–Davidson Resilience Scale (CD-RISC), the Spiritual Intelligence Self-Report Inventory (SISRI) and the Perceived Stress Scale (PSS) are used as a research instrument.

**Results:**

The results show that there is a positive and significant relationship between the SISRI and the CD-RISC. However, there is a negative and significant relationship between the SISRI and the PSS of students. The Enter regression analysis for prediction of the CD-RISC show that the SISRI predicts 0.10 of the CD-RISC variances and also the SISRI predicts 0.11 of the PSS variances.

**Conclusion:**

Spirituality helps to resilience in people who experience stress.

## Introduction

Psychologists have identified many areas of intelligence apart from the cognitive intelligence, such as fluid intelligence, crystallized intelligence, social intelligence, emotional intelligence, and spiritual intelligence; that all of them are indices of adjustment (1). Beardsley LM created the term spiritual intelligence and describe it as “an intelligence with which we address and solve problems of meaning and value, and it can place our actions and our lives in a wider, richer, meaning-giving context”. Furthermore by spiritual intelligence we can evaluate that one course of action is more meaningful than another (2).

Spiritual intelligence is concerned with the internal life of mind and spirit and its association with being in the world. It implies a capability for a deep understanding of existential questions and insight into multiple levels of consciousness. It is more than individual mental skill. In addition to self-awareness, it implies awareness of our relationship to the transcendent, to each other, to the earth and all beings (3). Spiritual intelligence is, therefore, an essential personal endowment which enables one to maintain both internal and external peace and display love regardless of the circumstances whether stress or acute inconsistency. It could, therefore, help in conflict management and calm co-existence in the society (1). Mascaro and Rosen have shown that if spiritual meaning is low, the relationship between stress and depression will be high, consequently suggesting that ability to create spiritual sense acts as a barrier against the effects of stress on well-being (4).

Positive psychology is a successful field of psychology, which includes the study of positive personality traits resilience, which is one of those characteristics (5), in psychology, is the positive capacity of people to deal with stress and adversity. This coping may result in the individual “bouncing back” to a previous state of normal functioning, or using the experience of facing adversity to produce a “steeling effect” and function better than expected (much like an inoculation gives one the ability to manage the future exposure to disease well (6).

According to Masten, resilience refers to successful adaptation of an individual in spite of risk and adversity (6). Singh and Yn argued that psychological resilience refers to effective coping and adaptation even though faced with the loss, hardship, or adversity (5). Carle and Chassin reported that individuals with high levels of self-reported resilience are particularly expected to use positive emotions to “bounce back” from adverse experiences (7). Empirical evidence suggests that resilience is grounded in a varied array of genetic, biological), psychological, and environmental factors (5). Although resilience resources can have a direct effect on health and well-being outcomes, the experience of stress (life events, perception of stress, stress exposure, and stress reactivity), the experience of stress is also likely to take on protective mechanisms in ways that influence individual life trajectories (physical health, functional status, subjective well-being, and psychological well-being) (8). Ahangar investigated resilience in reaction to life stress - the effects of coping style and cognitive power of endurance. In general, results supported a direct effects model of the relationship between life stress and psychological wellbeing. Results showed that the cognitive hardiness, aspects of coping style and negative life events directly impacted on measures of psychological and somatic suffering (9).

Therefore, resilience is a multidimensional construct that varies with context, time, age, and life conditions (5). The aim of this study was to examine the relationship between spiritual intelligence with resilience and the perceived stress. Accordingly the research questions are: 1) is there relationship between spiritual intelligence and resilience of university students? 2) Is there relationship between spiritual intelligence and perception of stress of university students?

## Materials and Methods

The sample was selected by clustering method. That is, at first the faculties were selected randomly and in the next step, the classes were chosen as clusters. The students filled in the questionnaires after their classes’ times if they were consent to do. Based on sampling Formula the sample size consists of 320 students that 13 questionnaires were omitted because of the deficiency and 307 one were used.

The research method was correlational descriptive. Statistical population was comprised of students of Sistan and Balochistan University.

The Pearson correlation and the step-wise analysis of regression methods were used for statistical analysis.

The Perceived Stress Scale (PSS) is the most widely used psychological instrument in measuring the perception of stress. It is a measure of the degree to which situations in one’s life are appraised a stressful. Cohen shows correlations with PSS and stress measures, self-reported health and health services measures, health behavior measures smoking status, help-seeking behavior. The PSS scores are obtained by reversing responses (e.g., 0 = 4, 1 = 3, 2 = 2, 3 = 1, and 4 = 0) to the four positively stated items (items 4, 5, 7, and 8) and then summing across all scale items. Coefficient alpha reliability for the PSS was 0.86 (10). In a sample of Iranian population, this coefficient was 0.81 (11).

The Resilience Scale for Adult is a scale with 43 items that by Friborg et al. proposed it by using factor analysis. Resilience scale has five dimensions of personal competence, social competence, social support, family coherence, and personal structure those measures the resilience in a 5-degree scale (12). Friborg et al. report the inner consistency, by using Cronbach alpha, for the scale 0.93 and for its subscales between ranges of 0.74-0.93 (12). Furthermore in Iran, Keshtkaran reported a Cronbach alpha for this scale as 0.93 and its subscales: personal competence as 0.83, social competence 0.86, social support 0.85, family coherence 0.84, and personal structure 0.76 (13).

The Spiritual Intelligence Self-Report Inventory (SISRI) is a 24-item scale that measures spiritual intelligent. Spiritual intelligence components are existential thinking, personal meaning production, transcendental awareness, and conscious state expansion. Sum of all item responses ranges from 0 to 96. Higher scores represent higher levels of spiritual intelligence. The SISRI displays excellent internal reliability (14). In a sample of Iranian population, the alpha coefficient was 0.89 (15).

## Results

Table 1 shows that there is a positive and significant relationship between the SISRI and the Connor–Davidson Resilience Scale (CD-RISC) of students.

**Table 1:** Correlation between resilience and spiritual intelligence

**Pearson correlation**	**Resilience**	**Spiritual intelligence**
**Resilience**	1.000	0.313*
**Spiritual intelligence**		1.000*

* P ≤ 0.010

In order to study the relationship of spiritual intelligence and perception of stress, the Pearson correlation and analysis of regression methods were used.

The amount of Durbin–Watson test shows that the regression analysis was suitable. This result shows that R and β of this relationship is 0.313. The finding also indicates that the SISRI predicts 0.10 of the CD-RISC variances (F = 32.475, df = 1, 298, p ≤ 0.01).

The results of table 2 show that there is a negative and significant relationship between the SISRI and the PSS of students.

The amount of Durbin–Watson test shows that the regression analysis was suitable. This result shows that R and β of this relationship is 0.110. The result also indicates that the SISRI predicts 0.11 of the PSS variances (F = 20.397, df = 1, 298, p ≤ 0.010).

**Table 2:** Correlation test between perception of stress and spiritual intelligence

**Pearson correlation**	**Stress of perception**	**Spiritual intelligence**
**Stress of perception**	1.000	−0.253*
**Spiritual intelligence**		1.000

* p ≤ 0.01

## Discussion

The purpose of this study was to investigate on relationship between spiritual intelligence, resilience, and perceived stress. The results of this study indicate that the spiritual intelligence has a positive association with resilience. This finding is consistent with researches that revealed components of resilience are hopefulness, personal control, coping, and religiosity/spirituality (8). On the other hand, spiritual intelligence includes neurological processes, particular cognitive capabilities and spiritual persona and interests (16). King that indicated spiritual intelligence’s capacity increase resilience and suggests that those with higher spiritual intelligence are more able to adapt and cope with difficulties by relying on internal strengths (14). Spiritual intelligence helps us to fight against problems of life and death and the deepest origins of human pain and despair (16). According to leading theorists, intelligence should smooth the progress in adjustment and problem-solving (17). Resilience also represents successful adaptation in the face of difficulties (18). In addition, the process of adaptation is affected by numerous factors and their connections in a person’s environment (18). Furthermore, Green and Noble reported the relationship between spirituality and resilience in the different cultural and religious backgrounds. Her study argued that spiritual experiences could lead to the ability to bear hardship (19). Spirituality has also been established to improve resilience among families suffering from parental loss (20). Therefore, spirituality helps to resilience in people who experience disease or disabilities. However, the intervention of resilience and spiritual intelligence on the perceived stress was the main aim of this study.

The results of the current study showed that spiritual intelligence and resilience have a negative relationship with perceived stress. There are few researches in this area. However, there are trustworthy findings on the remarkable degree of differences in the stress response (8). According to Lazarus and Folkman in addition to the situation, the individual’s assessment of the situational connection to well-being that potentially leads to a stress assessment (21). Therefore, an individual assess can make an event stressful and both individual factors and situational factors are needed to an event be appraised as stressful (21). Two judgments cause people to experience stress at the prospect of a performance situation: 1) their cognitive assessment of the difficulty of the situation and 2) their assessment of the resources that they have to cope with the situation (22). People assess the situation more threatening when they see their skills as falling short of the task demands, but if they believe they have the resources to meet the demands, they see it a challenge (23).

Therefore, spiritual intelligence allows us to reconsider our experiences and create meaning (17). Personal meaning production is an applicable component of spiritual intelligence (17). Studies in the resilience area also show that resilience is one of the important factors to lead a happy and healthy life, and it can increase efforts, especially under difficult situation (24). Cognitive theory has suggested many tools for developing more resilient. Dumont and Provost have showed that the resilient youngsters significantly use the positive coping strategy of problem-solving higher than other groups. Coping strategies focused on the problem-solving are aimed at doing something to change the stressful situation (25). Cognitive theory has suggested many tools for developing more resilient. Dumont and Provost have showed that the resilient youngsters significantly use the positive coping strategy of problem solving higher than other groups. Coping strategies focused on problem solving are aimed at doing something to change the stressful situation (25).

## Notes

**Declaration of interest:** None.
